# Distortion of Mendelian segregation across the Angus cattle genome uncovering regions affecting reproduction

**DOI:** 10.1038/s41598-023-37710-z

**Published:** 2023-08-17

**Authors:** S. Id-Lahoucine, J. Casellas, D. Lu, M. Sargolzaei, S. Miller, A. Cánovas

**Affiliations:** 1 Centre for Genetic Improvement of Livestock, Department of Animal Biosciences, University of Guelph, Guelph, ON N1G 2W1 Canada; 2 Departament de Ciència Animal i dels Aliments, Universitat Autònoma de Barcelona, Bellaterra 08193 Barcelona, Spain; 3 Angus Genetics Inc., St. Joseph, MO 64506 USA; 4 Department of Pathobiology, University of Guelph, Guelph, ON N1G 2W1 Canada; 5 Select Sires, Inc., Plain City, OH 43064 USA; 6 AGBU, a joint venture of NSW Department of Primary Industries and University of New England, Armidale, 2351 Australia

**Keywords:** Animal breeding, Genomics

## Abstract

Nowadays, the availability of genotyped trios (sire-dam-offspring) in the livestock industry enables the implementation of the transmission ratio distortion (TRD) approach to discover deleterious alleles in the genome. Various biological mechanisms at different stages of the reproductive cycle such as gametogenesis, embryo development and postnatal viability can induce signals of TRD (i.e., deviation from Mendelian inheritance expectations). In this study, TRD was evaluated using both SNP-by-SNP and sliding windows of 2-, 4-, 7-, 10- and 20-SNP across 92,942 autosomal SNPs for 258,140 genotyped Angus cattle including 7,486 sires, 72,688 dams and 205,966 offspring. Transmission ratio distortion was characterized using allelic (specific- and unspecific-parent TRD) and genotypic parameterizations (additive- and dominance-TRD). Across the Angus autosomal chromosomes, 851 regions were clearly found with decisive evidence for TRD. Among these findings, 19 haplotypes with recessive patterns (potential lethality for homozygote individuals) and 52 regions with allelic patterns exhibiting complete or quasi-complete absence for homozygous individuals in addition to under-representation (potentially reduced viability) of the carrier (heterozygous) offspring were found. In addition, 64 (12) and 20 (4) regions showed significant influence on the trait heifer pregnancy at p-value < 0.05 (after chromosome-wise false discovery rate) and 0.01, respectively, reducing the pregnancy rate up to 15%, thus, supporting the biological importance of TRD phenomenon in reproduction.

## Introduction

The agricultural industry has been revolutionized by the growing and extended use of DNA technologies, which can be attributed to the rapid development of high-throughput genotyping tools and the gradual decline of their costs. Since the introduction of commercial SNP array-based genotyping in the livestock industry, the use of genome-enabled prediction has significantly increased the rate of genetic progress^1–3^. In addition to the benefit of genomic evaluation on the prediction accuracy, the routine utilization of genotyping technologies on a daily basis provided a valuable resource of large and powerful data that can be explored for other innovative applications for animal breeding purposes. Particularly, the availability of large pedigree and consequently trios (i.e., sire-dam-offspring) of genotyped families enables the implementation of the transmission ratio distortion (TRD) approach, that could be viewed as an alternative and/or complementary method to discover more deleterious alleles affecting reproduction^4, 5^. It is known that lethal alleles are not transmitted according to Mendelian principles but deviated from inheritance expectations displaying signals of TRD in the genome^6, 7^. Despite a wide range of cases of known Mendelian disorders (Online Mendelian Inheritance in Animals (OMIA), 2012; https://omia.org/home/), large and dense genomic data provide opportunities to uncover more deleterious alleles by using genotypes of live animals without the need of affected individuals or phenotypes as other strategies do^8–10^. In livestock species, the method of approaching the whole genome using families of genotypes to screen for a specific TRD pattern (absence of homozygous haplotypes) in search of lethal alleles was started by VanRaden et al.^11^. More recently, Casellas et al.^4, 12–14^ and Id-Lahoucine et al.^5^ developed TRD models with allelic and genotypic parametrizations to identify genomic regions with multiple types of TRD (e.g., recessive, allelic, parent-specific).

Screening for deleterious alleles across the whole genome has been a successful strategy during the last decade^11, 15–19^, but large datasets are needed, as deleterious alleles typically exist with low frequencies in populations. Indeed, those rare variants are suggested to be functional, despite being more difficult to discover^20–22^. The increasing amount of genomic data contributes to overcoming this challenge and makes discovering genomic defects more feasible. To date, > 800,000 Angus animals have been genotyped in the last 10 years by Angus Genetics Inc. (S. P. Miller, American Angus Association, Saint Joseph, MO, personal communication). Taking into consideration that only 2 studies have been done using 3,993^23^ and 22,836^24^ Angus genotypes, the availability of this larger dataset (> 800,000) motivates the relevance of approaching lethal alleles with higher statistical power.

The objectives of our research were (1) characterization of the TRD phenomenon in Angus genome including the identification of deleterious alleles affecting reproduction and survival in Angus, (2) comparison of TRD findings across breeds between Angus and Holstein genomes, (3) examination of previously known lethal defects on Angus data, (4) discovering haplotypes that carry putatively lethal or semi-lethal alleles and (6) to quantify the effects of TRD regions on the heifer pregnancy trait.

## Methods

### Genotype data

In this study we used an Angus population with > 800,000 North American Angus animals genotyped as of November 2019. Only sample combining trios (sire-dam-offspring) of genotypes were kept for TRD analyses, thus, the dataset used consists of 258,140 genotypes from the Angus Genetics Inc. database (St. Joseph, MO, USA). The number of genotyped sires, dams and offspring were 7,486, 72,688 and 205,966, respectively. Animals were genotyped with different SNP genotyping arrays as part of ongoing commercial genotyping activities by breeders for genetic selection purposes. Commercial genotyping products were from Zoetis (i50K and HD50K (50 K SNPs; https://www.zoetisus.com/animal-genetics/beef/hd-50k/hd-50k-for-black-angus.aspx); Florham Park, NJ) and Neogen GeneSeek (GeneSeek Genomic Profile Low-Density (GGPLD; 40 K SNPs), High-Density (GGPHD; 80 K SNPs), GGPUHD (150 K SNPs; https://genomics.neogen.com/pdf/ag151_ggp_ts.pdf) and AngusGS (https://genomics.neogen.com/en/beef-cattle); Lincoln, NE). Both companies provided, for genomic evaluation purposes, an imputed SNP set similar to the Illumina 50KV2 (Illumina, Inc., San Diego, CA) and mapped to the ARS-UCD 1.2 genome assembly. All of the selected genotyped trios had a call rate > 90%. Additionally, autosomal SNPs with call rate > 90%, a minor allele frequency > 0.1% and a Mendelian inconsistency < 2% were selected for TRD analysis. For the purpose of imputation, FImpute^25^ (version 2.2) was used to impute and phase data for 92,942 SNPs. The imputation algorithm of FImpute takes advantage of the overlapping content between arrays and uses pedigree information to provide a more accurate imputation.

### Analytical models of transmission ratio distortion

#### Allelic parameterization of TRD

As described by Casellas et al.^4, 13^, for a particular locus, the probability of allele transmission (P) from heterozygote parents (A/B) to offspring was parameterized including one overall TRD effect (α) on a parent-unspecific model or differentiating between sire- (α_s_) and dam-specific TRD effects (α_d_) on a parent-specific model:

$${\text{P}}\left( {\text{A}} \right) \, = { 1 } - {\text{ P}}\left( {\text{B}} \right) \, = \, 0.{5 } + \, \alpha \,{\text{and}}\,{\text{P}}\left( {\text{B}} \right) \, = { 1 } - {\text{ P}}\left( {\text{A}} \right) \, = \, 0.{5 } - \, \alpha ,$$

$${\text{P}}_{{\text{i}}} \left( {\text{A}} \right) \, = { 1 } - {\text{ P}}_{{\text{i}}} \left( {\text{B}} \right) \, = \, 0.{5 } + \, \alpha_{{\text{i}}} \,{\text{and}}\,{\text{ P}}_{{\text{i}}} \left( {\text{B}} \right) \, = { 1 } - {\text{ P}}_{{\text{i}}} \left( {\text{A}} \right) \, = \, 0.{5 } - \, \alpha_{{\text{i}}} \,{\text{with}}\,{\text{i }} = \, \left[ {{\text{s}},{\text{d}}} \right]$$

Flat priors (uniform distribution) were assumed for all TRD parameters within a parametric space ranging from − 0.5 to 0.5. Under a Bayesian implementation, the conditional posterior probabilities of the TRD parameters are defined as:

$${\text{p}}\left( {\alpha |{\mathbf{y}}} \right) \propto {\text{p}}\left( {{\mathbf{y}}|\alpha } \right){\text{p}}\left( \alpha \right){\text{ and p}}\left( {\alpha_{{\text{s}}} ,\alpha_{{\text{d}}} |{\mathbf{y}}} \right) \propto {\text{p}}\left( {{\mathbf{y}}|\alpha_{{\text{s}}} ,\alpha_{{\text{d}}} } \right){\text{p}}\left( {\alpha_{{\text{s}}} } \right){\text{p}}\left( {\alpha_{{\text{d}}} } \right)$$

where; **y** is the column vector of genotypes of the offspring generation. The likelihood of data is a multiplication of the corresponding probabilities for each offspring as:

$$\mathrm{p}\left(\mathbf{y}|\mathrm{\alpha }\right)={\prod }_{\mathrm{n}}{\mathrm{P}}_{\mathrm{off}}({\mathrm{y}}_{\mathrm{i}})\,{\text{ and p}}\,(\mathbf{y}|{\mathrm{\alpha }}_{\mathrm{s}},{\mathrm{\alpha }}_{\mathrm{d}})={\prod }_{\mathrm{n}}{\mathrm{P}}_{\mathrm{off}}({\mathrm{y}}_{\mathrm{i}})$$

where *n* is the total number of offspring and P_off_ and y_i_ is the probability and the genotype of the *i*_th_ offspring, respectively. The probability of the genotype of each offspring was defined by parents’ genotypes and TRD parameters. Thus, the probability of a heterozygous offspring from a heterozygous-by-heterozygous mating becomes:

$${\text{P}}_{{{\text{off}}}} \left( {{\text{AB}}} \right) \, = \, [(0.{5 } + \alpha_{{\text{s}}} )(0.{5 } - \alpha_{{\text{d}}} )\left] { \, + \, } \right[(0.{5 } - \alpha_{{\text{s}}} )(0.{5 } + \alpha_{{\text{d}}} )]$$

Detailed information about the implemented algorithms were described in Id-Lahoucine et al.^5^ and Id-Lahoucine^26^.

#### Genotypic parameterization of TRD

As developed by Casellas et al.^12^, genotypic parameterization can be modeled by assuming additive (α_g_) and dominance (δ_g_; or over- / under-dominance) parameters, regardless of the origin of each allele. Following Casellas et al.^14^, the probability of the offspring (P_off_) from heterozygous-by-heterozygous mating are:

$${\mathrm{P}}_{\mathrm{off}}\left(\mathrm{AA}\right)=\frac{\left(1+{\mathrm{\alpha }}_{\mathrm{g}}-{\updelta }_{\mathrm{g}}\right)}{4}, {\mathrm{P}}_{\mathrm{off}}\left(\mathrm{AB}\right)=\frac{\left(1+{\updelta }_{\mathrm{g}}\right)}{2}\text{ and }{\text{P}}_{\mathrm{off}}\left(\mathrm{BB}\right)=\frac{\left(1-{\mathrm{\alpha }}_{\mathrm{g}}-{\updelta }_{\mathrm{g}}\right)}{4}$$

For heterozygous-by-homozygous mating, correction for overall losses of individuals in terms of genotypic frequency are needed to guarantee P_off_(AA) + P_off_(AB) + P_off_(BB) = 1. Thus, genotypic frequencies in offspring from AA × AB mating as an example become:

$${\mathrm{P}}_{\mathrm{off}}\left(\mathrm{AA}\right)=\frac{\left(1+{\mathrm{\alpha }}_{\mathrm{g}}-{\updelta }_{\mathrm{g}}\right)}{2\times (1+{\mathrm{\alpha }}_{\mathrm{g}}/2)}, {\mathrm{P}}_{\mathrm{off}}\left(\mathrm{AB}\right)=\frac{\left(1+{\updelta }_{\mathrm{g}}\right)}{2\times (1+{\mathrm{\alpha }}_{\mathrm{g}}/2)}\text{ and }{\text{P}}_{\mathrm{off}}\left(\mathrm{BB}\right)=0$$

Under a Bayesian implementation, the conditional posterior probabilities of the TRD parameters are defined as:

$${\text{p}}\left( {\alpha_{{\text{g}}} ,\delta_{{\text{g}}} |{\mathbf{y}}} \right) \propto {\text{p}}\left( {{\mathbf{y}}|\alpha_{{\text{g}}} ,\delta_{{\text{g}}} } \right){\text{p}}\left( {\alpha_{{\text{g}}} } \right){\text{p}}\left( {\delta_{{\text{g}}} |\alpha_{{\text{g}}} } \right)$$

Flat priors were assumed for both α_g_ and δ_g_ within a deepened parametric space (i.e., the parametric space of a parameter is conditioned to the other parameter). Thus, the parameter space for α_g_ initially ranges [− 1, 1] with a p(α_g_) = ½ and becomes conditioned to δ_g_ when δ_g_ > 0, being restricted to [− 1 + δ_g_, 1 − δ_g_] with a p(α_g_) = 2/(2–2 × δ_g_). On the other hand, the parametric space for δ_g_ competent ranges [− 1, |α_g_|] with a p(δ_g_) = 1/(1 + α_g_). Notice that these conditions were made to avoid negative probabilities for a given offspring from a particular mating.

### Statistical analyses

The analyses of TRD were evaluated SNP-by-SNP and using a sliding windows approach for haplotypes of 2-, 4-, 7-, 10- and 20-SNP across 92,942 SNPs. For haplotype analyses, the biallelic-haplotype procedure described by Id-Lahoucine et al.^5^ was implemented following the same parameterization described above. The analyses were performed within a Bayesian framework using TRDscan v.1.0 software^5^ with a unique Monte Carlo Markov chain of 110,000 iterations where the first 10,000 iterations were discarded as burn-in. The statistical relevance of TRD was evaluated using a Bayes factor^27^ (BF). The BF estimates was obtained across iterations with a lag interval of 10 iterations. Both allelic and genotypic parameterizations were compared using the deviance information criterion^28^ (DIC). In order to optimize the TRD analyses, the following steps were considered following Id-Lahoucine et al.^5^. Firstly, a minimum of 1,000 informative offspring was considered to guarantee minimal statistical power to characterize TRD across the whole genome. Secondly, a minimum number of informative parents (≥ 20 heterozygous sires and/or ≥ 100 heterozygous dams) were considered to minimize possible false TRD from genotyping errors. As post analyses criteria, the approximate empirical null distribution of TRD^5^ at < 0.001% margin error was applied in order to exclude TRD generated by chance (i.e., gametes sampling fluctuations). In the same way, regions with few heterozygous sires displaying full skewed transmission and completely explaining the observed TRD in the corresponding region were discarded as potential genotyping errors. Subsequently, regions with a large credible interval for TRD effects (i.e., coefficient of variation > 20%), potentially as a result of unstable convergence, were filtered out. Finally, in order to combine and integrate all the results to obtain clear highlighted peaks of TRD across the whole genome, the kernel smoothing^29, 30^ (parametric technique) was applied. The smoothed estimate of BF for the ith base pair (bp) within the range κ_i_ to κ_n_, was calculated using weighted Gaussian kernel (${\widehat{\mathrm{y}}}_{\mathrm{i}}=\sum_{\mathrm{j}=1}^{\mathrm{n}}\frac{1}{\sqrt{2{\mathrm{\pi \sigma }}^{2}}}\mathrm{exp}\left(-\frac{{({\mathrm{k}}_{\mathrm{i}}-{\mathrm{k}}_{\mathrm{j}})}^{2}}{2{\upsigma }^{2}}\right)\times {\mathrm{BF}}_{\mathrm{j}}$), where σ is the bandwidth and (κ_i_—κ_j_) is the distance in base pairs. Following Id-Lahoucine^26^, the smoothing process was implemented with a bandwidth of 500,000 bp, which is suggested to be a rationale distance to obtain a considerable initial number of candidate regions in TRD analyses.

### Characterization of TRD effects on reproductive phenotypes

As an additional analyses, the effects of TRD regions (SNPs or haplotypes) were evaluated using the heifer pregnancy trait as recorded in the whole American Angus database. To determine the effects of the alleles, pregnancy rate between matings at risk and control were compared in two ways (for each region separately): AB × AB (risk) with AA × – (control) and AB × – (risk) with AA × AA (control). This first comparison allows to determine the impact of recessive TRD regions whereas the second is useful for allelic TRD regions. The rationale behind these matings is that we do not expect to observe BB offspring for recessive TRD regions, thus, both heterozygous parents are needed for the test. On the other hand, the presence of one single heterozygous parent is enough for testing allelic TRD regions as AB offspring could also present reduced viability. This interaction effect was included in the following animal model:

$${\text{PHN}} = {\text{ INT }} + {\text{ CG }} + {\text{ ADH }} + {\text{ HA }} + {\text{ SS }} + {\text{ A }} + {\text{e}}$$

where; PHN was the phenotypic recorded as binary traits (i.e., pregnant or not pregnant), INT is the interaction effect between parent genotypes (recorded as 1 and 0 for mating at risk and control, respectively), CG is a contemporary group (fixed effect comprised of the unique combination of herd-breeding year-season-breeding group-synchronization), ADH is the age the heifer’s dam (fixed effect), HA is heifer age at breeding (covariate), SS is first service sire (random effect), A is the animal additive genetic effect and e is the random residual term. The effects included in the model are similar to those used in the national genetic evaluation of American Angus (Angus Genetics Inc., St. Joseph, MO, USA). The analysis was performed using a linear model (assuming Gaussian distribution for random effects). The animal additive genetic follows a multivariate normal distribution, i.e., MVN(0, **G**σ_a_^2^), where σ_a_^2^ was the genetic variance and** G** was the genomic relationship matrix constructed with 88,959 SNPs (minor allele frequency > 0.001) using VanRaden’s first method^31^. The significance of the interaction effect was tested with a t-test. The total number of genotyped heifers with a pregnancy record was 21,297. The total number of pregnancy records where at least one parent is genotyped was 70,869. When considering the maternal grandsire genotype (i.e., the sire of the heifer), the number of informative records increased to 76,719.

### Ethics declarations

Data used in this research were obtained from commercial producer, thus, animal care approval was not required.

## Results and discussion

### Characterization of TRD on Angus genome

Single nucleotide polymorphism and haplotype alleles were identified exhibiting distorted segregation ratios with decisive evidence (BF ≥ 100 according to Jeffreys’ scale^32^) across the Angus genome. After the implementation of the different strategies to minimize possible TRD artifacts, a total of 99,580 genomic regions with TRD were identified (including totally or partially overlapped windows) after exclusively keeping the allele-region (SNP or the haplotype allele) with the highest BF. Among them, 5,027 corresponded to SNPs and 9,913, 14,106, 18,088, 21,368 and 31,078 corresponded to haplotype windows of 2-, 4-, 7-, 10- and 20-SNP, respectively. This large number was a result of the sliding window approach, the different window sizes applied and the level of linkage disequilibrium (LD). Thus, it is important to mention that the signals of TRD observed for individual SNPs and/or short haplotype windows are also observed in windows linked to them. Given that the different TRD patterns observed across adjacent regions were potentially generated from one single mutation. Here, we assumed that the best candidate allele harboring the causal variant (or in strong LD with it) will correspond to the allele-region with the highest BF^5^. Thus, after combining and integrate all the results taking into account the LD using the smoothing process, 990 core regions has been highlighted across the whole genome. Within these regions, 139 regions were excluded as they were plausibly explained by genotyping errors or convergence instability after individually checking (visual inspection) the mean and standard deviation of TRD parameters and the corresponding distribution of the offspring across matings. Notice that for genotyping errors could be anticipated when checking the number of heterozygous sires (with at least 10 offspring) that transmitted one allele with a probability > 90% and the distribution of their offspring. Following Id-Lahoucine et al.^5^, the strategy used is based on discarded TRD that was generated fully from few heterozygous sires (e.g., < 3) with a large number of offspring, these sires potentially are homozygous and genotyped incorrectly as heterozygous.

### Relevant insight of TRD findings on Angus genome with deleterious alleles.

The whole Angus genome was characterized with 851 non-overlapping TRD regions, being 177 SNPs and 258, 165, 103, 78 and 70 haplotype windows of 2-, 4-, 7-, 10- and 20-SNP, respectively. Among these findings, it is important to highlight that the majority of regions were detected with more than one of the applied models (i.e., parent-unspecific and -specific allelic model, genotypic model). Despite this overlap, different statistical evidence was observed for TRD estimates for the different models, suggesting different degrees of fit, and consequently, distinctive patterns of inheritance.

#### Allelic patterns

**Figure 1:**
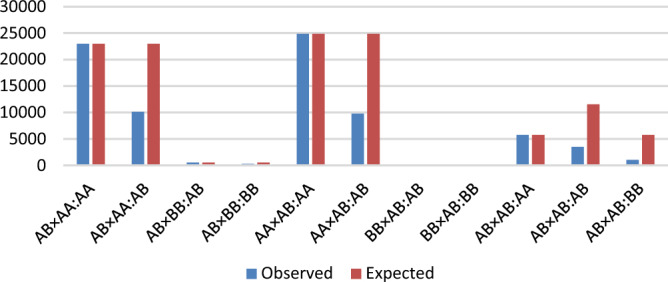
Number of observed and expected offspring for each sire and dam mating and offspring genotypes (sire × dam:offspring) of the TRD region with the highest number of under-repented offspring on the Angus genome. Individual SNP with an overall TRD = 0.21 and log_10_(BF) = 3,553.34.

**Figure 2:**
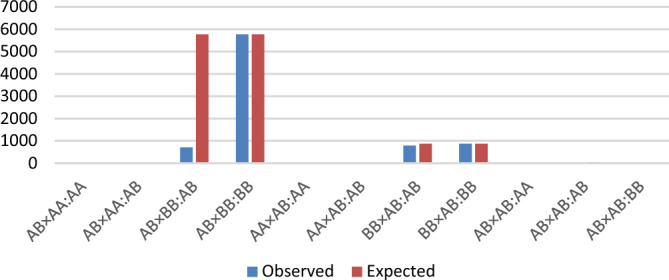
Number of observed and expected offspring for each sire and dam mating and offspring genotypes (sire × dam:offspring) of the region with sire-TRD on the Angus genome. Haplotype allele with sire-TRD =  − 0.39 and log_10_(BF) = 978.93 and dam-TRD =  − 0.02 and log_10_(BF) =  − 0.64.

The majority of TRD regions (657) presented an allelic pattern (i.e., identified with the allelic model with strong relevance). Loci with parent-specific TRD were 3 and 131 for dam- and sire-TRD, respectively. In order to target the most promising regions, following Id-Lahoucine^26^, a moderate-to-high TRD of > 0.20 was considered with at least 5,000 under-represented offspring. That is, 52 regions were selected as the most relevant (Table 1 (first 20 regions) and S1 Table (full list)). The average number of under-represented offspring across 52 regions was 10,099, being 41,008 the maximum number of under-represented offspring (Fig. 1). This finding shows that under the hypothesis of lethality, potentially 41,008 offspring would be lost given one single deleterious allele. This particular region was found with 79,200 informative offspring and a frequency of 0.08 (corresponding to the haplotype allele that is under-transmitted). In addition, among these regions, the penetrance (TRD magnitude) via sire and dam was equal or slightly different in 51 TRD regions (S1 Table). In contrast, only one single region exhibited sire-specific TRD whereas was null via dam (Fig. 2). It is important to add, even those regions had moderate-to-high TRD signals, part of them may have TRD linked to specific families and where further research is required to better target the causal mutation.

**Table 1:** Potential candidate lethal or semi-lethal haplotype alleles with allelic transmission ratio distortion (TRD) patterns (allelic parametrization) in Angus cattle.

Region	Window size^a^	Number of heterozygous sires	Number of heterozygous dams	Frequency	Overall TRD	Log_10_ (BF)	Sire-TRD	Log_10_ (BF)	Dam-TRD	Log_10_ (BF)	Number of under-repented offspring
AA.1	1	216	1508	0.991	0.27	969	0.31	927	0.17	106	7,897
AA.2	4	109	883	0.009	− 0.24	739	− 0.27	805	− 0.07	10	6,953
AA.3	1	103	906	0.997	0.42	1088	0.43	497	0.4	590	5,212
AA.4	7	71	615	0.002	− 0.48	1505	− 0.49	949	− 0.46	563	5,704
AA.5	1	136	1349	0.006	− 0.4	2557	− 0.4	1678	− 0.4	867	12,834
AA.6	1	162	1309	0.006	− 0.3	827	− 0.28	395	− 0.32	428	6,004
AA.7	2	180	1823	0.01	− 0.25	835	− 0.24	457	− 0.28	375	7,275
AA.8	2	108	753	0.002	− 0.46	1357	− 0.48	570	− 0.46	785	5,395
AA.9	2	670	4497	0.038	− 0.22	2010	− 0.25	1890	− 0.13	175	20,615
AA.10	2	115	939	0.003	− 0.41	1261	− 0.45	1032	− 0.33	273	6,191
AA.11	1	352	3655	0.017	− 0.25	1124	− 0.32	844	− 0.19	341	9,888
AA.12	10	38	364	0.004	− 0.32	762	− 0.39	979	− 0.02	− 1	5,169
AA.13	1	136	1367	0.004	− 0.41	1469	− 0.44	885	− 0.37	591	7,412
AA.14	4	47	497	0.001	− 0.47	1385	− 0.49	982	− 0.44	418	5,371
AA.15	7	116	1213	0.004	− 0.4	1332	− 0.42	875	− 0.36	463	6,782
AA.16	10	624	5405	0.031	− 0.24	1629	− 0.34	1652	− 0.14	235	14,985
AA.17	7	166	932	0.01	− 0.26	1025	− 0.27	993	− 0.17	46	8,713
AA.18	1	363	2512	0.014	− 0.28	1616	− 0.3	1023	− 0.25	565	12,803
AA.19	7	85	998	0.004	− 0.37	1123	− 0.43	1015	− 0.26	192	6,240
AA.20	4	94	618	0.002	− 0.45	2019	− 0.47	1697	− 0.39	350	8,404

^a^Number of SNPs on the window; ^b^Parents’ genotypes; ^c^Offpsring genotype; BF: Bayes factor; Full list of regions is provided in S1 Table.

On the other hand, it is very interesting to mention that one single region of the allelic pattern was observed with opposite sire- and dam-TRD, where sires showed a preferential transmission of one allele (3,055 AB and 1,014 BB offspring from AB (sire) × BB mating) whereas dams showed a preferential transmission for the opposite allele (1,074 AB and 1,870 BB offspring from BB × AB (dam) mating). In fact, an opposite sire- and dam-TRD were also observed on other regions displaying an excess or deficiency of heterozygous offspring (e.g., sire-TRD = − 0.03, dam-TRD = 0.04, 14,406 AA, 16,618 AB and 10,300 BB from AB × AB mating), but this remarkable region showed a peculiar pattern which adds complexity to TRD phenomenon in cattle.

#### Genotypic patterns

The genotypic model highlighted 19 regions with recessive patterns (Table 2) and 9 with either deficiency or excess of heterozygous offspring. Here, a minimal 10 ≥ non-observed homozygous offspring was required to target recessive TRD. Thus, the number of non-observed homozygous offspring for these regions with recessive pattern ranges from 10 to 564. The lethality among these regions was diverse, some with potentially full lethality (i.e., full absence of homozygous haplotype) or with reduced viability of offspring in homozygous state. Here, 9 haplotypes were detected with full absence of homozygous offspring. The lethality on other regions was observed with different degrees, comprising the smallest change of mortality to 40%. For an illustrative example, a specific region (AR.9) with 176 AA, 1,528 AB and 740 BB offspring from AB × AB matings had an anticipated rate of mortality of 76% ((740–176)/740*100)); notice that 1,528 (AB) / 740 (BB) ≈ 2 maintains the expected Mendelian ratio. It is important to consider that for this pattern the reduced viability was observed only on homozygous offspring and not heterozygous offspring as in the case of allelic TRD patterns.

**Table 2:** Potential candidate lethal or semi-lethal haplotype identified with recessive transmission ratio distortion (TRD) patterns (genotypic parametrization) in Angus cattle.

Region^a^	Window size	Number of heterozygous sires	Number of heterozygous dams	Frequency	Additive-TRD	Log_10_ (BF)	Dominance-TRD	Log_10_ (BF)	AB × AB		
									AA	AB	BB
AR.1	20	223	2092	0.014	− 0.66	271.0	0.31	140.1	0	100	51
AR.2	20	99	473	0.006	− 0.79	73.9	0.09	2.7	0	19	12
AR.3	2	46	748	0.005	− 0.72	105.3	0.18	18.7	0	21	14
AR.4	20	103	1045	0.006	− 0.75	71.7	0.15	8.7	0	25	12
AR.5	7	215	2166	0.013	− 0.44	95.9	0.18	44.7	8	54	28
AR.6	20	89	696	0.006	− 0.60	82.4	0.29	44.0	0	18	12
AR.7	20	45	508	0.005	− 0.63	88.4	0.26	37.9	0	24	11
AR.8	20	95	735	0.004	− 0.57	53.8	0.33	37.3	0	22	14
AR.9	20	642	5279	0.054	− 0.48	595.2	0.23	347.2	176	1528	740
AR.10	1	1114	10,715	0.071	− 0.24	184.2	0.12	129.7	599	2186	1029
AR.11	20	116	668	0.007	− 0.48	72.9	0.25	50.4	4	43	20
AR.12	20	309	2884	0.025	− 0.52	295.2	0.25	171.2	10	108	61
AR.13	4	419	4101	0.029	− 0.28	99.6	0.14	74.7	79	335	138
AR.14	4	427	4105	0.030	− 0.28	109.1	0.15	82.9	88	378	150
AR.15	7	445	4528	0.033	− 0.21	61.7	0.11	56.7	121	389	209
AR.16.a	4	339	3432	0.028	− 0.64	598.2	0.32	331.9	4	473	216
AR.16.b	20	263	2820	0.027	− 0.64	553.4	0.35	343.1	0	436	187
AR.16.c	4	351	3493	0.028	− 0.40	219.7	0.21	155.2	71	438	208
AR.17	20	194	1787	0.011	− 0.55	132.5	0.28	80.6	3	56	33
AR.18	20	328	3423	0.020	− 0.50	190.5	0.24	112.8	16	161	76
AR.19	20	82	639	0.004	− 0.54	32.5	0.25	17.7	0	22	11

^a^Number of SNPs on the window; ^b^Parents’ genotypes; ^c^Offpsring genotype; BF: Bayes factor; Full list of regions is provided in S2 Table.

On the other hand, for the detected regions with recessive patterns, 3 physically close haplotypes (AR.16.a, AR.16.b and AR.16.c; covering 11,925 Kbp;) showed similar TRD magnitudes, frequencies and number of heterozygous sires and dams (Table 2), which potentially points to the same causal mutation (SNP, deletion, etc.). The LD between these 3 haplotype alleles (biallelic-haplotype genotypes) were 0.76, 0.43 and 0.51. This result gives extra evidence supporting the TRD found in this particular region.

### Model comparison

The DIC values across models supported the inheritance pattern of TRD region described in the previous sections. Specifically, for recessive TRD regions the genotypic model was favored in comparison to the allelic model with differences up to 209.40 DIC units (average across the 19 regions was 38.99). In contrast, among the regions with allelic pattern (52), 49 fit better for the allelic model, displaying deference of DIC units ranging up to 630.76 with an average of 116.07 DIC units. The remaining regions (3 from 52), despite displaying low DIC for the genotypic model, the distribution of offspring across matings presented an allelic pattern. Their DIC advantage was coming from the combination of additive and dominance parameters that maximizes the likelihood of data, resulting in a similar or better fit for both models.

### Validation and comparison of TRD phenomenon and lethality across breeds.

In general, when comparing TRD findings between Angus and Holstein breeds^26^, the observed prevalence and magnitude of TRD were higher in Angus population. Whereas the number of regions in Angus was 851 with an average of 0.27 overall TRD magnitude, the number of regions identified in another study from our group in Holstein genome was 604 with an average of 0.22 TRD magnitude^26^. In relation to statistical evidence, 814 and 560 regions presented a log_10_(BF) ≥ 10 for overall TRD for Angus and Holsteins, respectively. It is important to mention that this is not a limitation of statistical power because the number of trios used in Holsteins (283,791) was even slightly superior to Angus (205,954). The observed differences between breeds could be explained partially by the different genotype density used for TRD analyses in both breeds, where higher density SNP array was used in Angus (92,942 SNPs) compared to Holsteins (47,910 SNPs). The advantage of using high-density genotypes, which enables the whole genome to be explored more deeply, allows the potential discovery of more candidate deleterious alleles.

On the other hand, similar patterns of overall and sire-TRD were observed in both Angus and Holstein breeds in similar positions across the genome. Among the 851 and 604 characterized TRD regions in Angus and Holsteins, respectively, 353 regions presented similar allelic TRD patterns with 46 of them being specific sire-TRD. Regarding the recessive TRD pattern, only one single region identified in Angus with recessive pattern was physically close to a known lethal allele located in BTA21:21,184,869–21,188,198 (AR.16, Table 2) in Holstein cattle with recessive inheritance as well (Holstein haplotype 0^33, 34^). The causative mutation in Holstein haplotype 0, responsible for the brachyspina syndrome, was a 3.3 Kb deletion in the FA complementation group I (*FANCI*) gene^34^. If we assume no recent common ancestor between both breeds, it is probably the results of independent mutations in the same genes which generated similar TRD patterns in both breeds, and consequently, may support the biological function of those genes on reproduction-related traits.

Within the same context, one of the detected regions with recessive TRD pattern overlapped with a previously reported candidate lethal allele by Jenko et al.^24^ located in BTA14:8,064,004–8,927,881 (AR.11, Table 2) in Aberdeen Angus. This reported haplotype was found to be associated with decreased insemination success and longer interval between insemination and calving^24^. The candidate gene for this haplotype was Zinc finger and AT-hook domain containing (*ZFAT*) which is associated with prenatal or perinatal lethality in the Mouse Informatics Database^24^. In addition, previously characterized lethal alleles by Jenko et al.^24^ in Simmental (BTA13:73,746,516–74,973,171) and Limousin (BTA23:27,923,154–28,649,349) were also physically overlapping with our findings, specifically among the relevant 52 allelic TRD regions (AA.33 and AA.44, S1 Table). On the other hand, among the 7 recessive lethal haplotypes reported by Hoff et al.^23^ in Angus, 3 were found overlying with our results in our Angus data but displaying allelic patterns: BTA8:62,040,920–63,000,189 (AA.21), BTA1:27,786,985–29,095,768 (AA.2) and BTA4:82,467,969–83,996,686 (AA.14). Hoff et al.^23^ identified a candidate gene located in BTA1, glycogen branching enzyme *(GBE1)*, which found to produce recessive phenotypes in mammals.

### Validation of the identified TRD regions using reproductive phenotypes: heifer pregnancy trait

Significant effects of TRD were found in the heifer pregnancy data. In total, 64 and 20 regions showed significant effects at p-value < 0.05 and 0.01, respectively. Particularly, when comparing between AA × AA and AB × – (mating risk), 49 and 12 regions displayed significant effect for the interaction effect on parent genotypes (at p-value < 0.05 and 0.01, respectively; Table 3 (first 30 regions) and S2 Table (full list)). The number of significant regions after controlling false discovery rate (FDR) at chromosome level^35, 36^ was 8 and 2 (at q-value < 0.05 and 0.01, respectively). The maximum observed effect was − 0.085. Hence, whereas for non-risk mating (i.e., AA × AA) the average pregnancy rate was 0.87, the observed pregnancy rate reduced down to as low as 0.74, that is, 15% reduced the pregnancy rate. It is important to mention that these effects were supported by the distribution of the pregnancy rate among both sire × dam and sire × maternal grandsire matings. The use of sire × maternal grandsire matings, allows increasing the number of informative matings by using phenotypes of non-genotyped heifers. These results support the relevance of the allelic TRD pattern, where the presence of the deleterious allele in one single parent is enough to reduce the pregnancy success of the animals. In addition, among these 49 regions, only 3 regions (Reg.12 (AA15), Reg.21 (AA.24) and Reg.44 (AA.50)) presented high TRD magnitude (> 0.20) and exhibiting more than 5,000 under-represented offspring. However, the average TRD magnitude and the number of under-represented among the 49 regions significant with the heifer pregnancy was 0.32 and 2,608, respectively (Table 3 and S2 Table).

**Table 3:** Effects of transmission ratio distortion (TRD) regions on heifer pregnancy and distribution of pregnancy among at risk (one or both parents carrying the deleterious allele) and control matings.

Mating type	Effect on phenotype	P-value (FDR)	Sire × dam genotypes		Sire × maternal grandsire genotypes		Overall-TRD (Log_10_ (BF)	Under-represented offspring
Region			AA × AA^b^ (^c^)	AB × –	AA × AA	AB × –		
Reg.1	− 0.034	0.013 (0.663)	0.869 (20,903)	0.769 (195)	0.855 (56,319)	0.801 (593)	− 0.46 (292.39)	1192
Reg.2	− 0.039	0.0225 (0.574)	0.87 (20,716)	0.868 (408)	0.854 (56,246)	0.879 (594)	− 0.39 (351.91)	1832
Reg.3	− 0.036	0.0402 (0.804)	0.87 (20,739)	0.808 (772)	0.855 (55,780)	0.817 (1260)	− 0.38 (226.68)	1238
Reg.4	− 0.037	0.0384 (1.535)	0.87 (20,744)	0.835 (1936)	0.855 (54,321)	0.84 (3604)	− 0.4 (597.74)	2953
Reg.5	− 0.034	0.0036 (0.155)	0.87 (20,720)	0.851 (1642)	0.855 (54,614)	0.85 (2860)	− 0.39 (317.23)	1687
Reg.6	− 0.026	0.0308 (0.295)	0.871 (20,252)	0.769 (1116)	0.856 (55,275)	0.791 (1591)	− 0.18 (69.44)	899
Reg.7	− 0.043	0.0113 (0.272)	0.87 (20,701)	0.838 (735)	0.855 (55,797)	0.826 (1155)	− 0.33 (396.98)	2530
Reg.8	− 0.035	0.0203 (0.244)	0.87 (20,650)	0.833 (424)	0.855 (56,300)	0.844 (646)	− 0.15 (49.41)	775
Reg.9	− 0.049	0.0088 (0.421)	0.87 (20,740)	0.846 (311)	0.855 (56,329)	0.851 (578)	− 0.41 (364.2)	1780
Reg.10	− 0.03	0.0177 (0.284)	0.87 (20,825)	0.778 (342)	0.855 (56,408)	0.769 (459)	− 0.38 (192.28)	1044
Reg.11	− 0.033	0.0444 (0.843)	0.87 (20,706)	0.825 (762)	0.855 (55,758)	0.825 (1191)	− 0.43 (634.78)	2826
Reg.12 (AA.15)	− 0.02	0.0105 (0.399)	0.87 (19,953)	0.854 (2838)	0.854 (53,043)	0.856 (4368)	− 0.4 (1331.99)	6782
Reg.13	− 0.074	0.0006 (0.025)	0.87 (20,781)	0.796 (285)	0.855 (56,473)	0.814 (365)	− 0.39 (212.15)	1123
Reg.14	− 0.036	0.0447 (0.581)	0.869 (20,753)	0.822 (1143)	0.856 (55,549)	0.814 (1575)	− 0.47 (636.15)	2501
Reg.15	− 0.055	0.0131 (0.256)	0.87 (20,806)	0.787 (643)	0.855 (55,958)	0.81 (1062)	− 0.35 (123.95)	754
Reg.16	− 0.045	0.0172 (0.189)	0.869 (20,748)	0.838 (2167)	0.854 (53,876)	0.844 (3387)	− 0.38 (156.3)	862
Reg.17	− 0.01	0.0141 (0.233)	0.872 (14,877)	0.854 (14,586)	0.853 (39,335)	0.852 (20,842)	− 0.03 (55.54)	4431
Reg.18	− 0.036	0.0126 (0.415)	0.87 (20,596)	0.811 (647)	0.855 (56,048)	0.823 (879)	− 0.33 (303.55)	1933
Reg.19	− 0.039	0.0319 (0.426)	0.87 (20,750)	0.821 (302)	0.855 (56,431)	0.832 (440)	− 0.2 (87.71)	999
Reg.20	− 0.036	0.008 (0.321)	0.87 (20,878)	0.835 (194)	0.855 (56,533)	0.848 (290)	0.36 (414.62)	2411
Reg.21 (AA.24)	− 0.025	0.025 (0.5)	0.87 (20,715)	0.867 (828)	0.855 (55,873)	0.871 (1331)	0.44 (1344.58)	5957
Reg.22	− 0.024	0.0144 (0.144)	0.872 (20,069)	0.813 (1283)	0.856 (55,245)	0.819 (1601)	− 0.41 (609.29)	3002
Reg.23	− 0.081	0.0056 (0.169)	0.87 (20,659)	0.742 (190)	0.855 (56,427)	0.74 (331)	− 0.46 (341.54)	1387
Reg.24	− 0.057	0.0129 (0.194)	0.87 (20,818)	0.81 (849)	0.856 (55,082)	0.816 (1546)	− 0.47 (281.53)	1116
Reg.25	− 0.023	0.0276 (0.995)	0.869 (20,298)	0.855 (1782)	0.855 (54,692)	0.852 (2660)	− 0.41 (893.66)	4367
Reg.26	− 0.023	0.0342 (0.616)	0.872 (20,246)	0.822 (1421)	0.856 (54,933)	0.826 (2149)	− 0.11 (71.16)	1520
Reg.27	− 0.036	0.0336 (0.521)	0.87 (20,729)	0.818 (352)	0.855 (56,216)	0.819 (656)	− 0.2 (87.25)	975
Reg.28	− 0.055	0.0001 (0.003)	0.871 (20,534)	0.803 (753)	0.856 (55,785)	0.811 (1199)	− 0.45 (549.38)	2324
Reg.29	− 0.06	0.0032 (0.041)	0.87 (20,764)	0.855 (574)	0.855 (56,057)	0.834 (808)	− 0.38 (623.83)	3337
Reg.30	− 0.079	0.001 (0.025)	0.87 (20,818)	0.821 (476)	0.855 (56,096)	0.82 (802)	− 0.16 (65.27)	929

^a^Number of SNPs on the window; ^b^Parents’ genotypes; ^c^Number of matings; FDR: chromosome-wise false discovery rate; Full list of regions is provided in S2 Table.

On the other hand, for AB × AB risk mating (recessive pattern), 15 and 8 regions displayed significant effect for the interaction effect on parent genotypes (at p-value < 0.05 and 0.01, respectively; Table 4). After chromosome-wise FDR, the number of regions reduced to 4 and 2 (at q-value < 0.05 and 0.01, respectively). The region (Reg. 52; Table 4) with the largest observed effect was − 0.27, with a pregnancy rate of 0.58 (corresponding to the 31% reduced the pregnancy rate) but with only 9 informative records, using maternal grandsire matings, the observed pregnancy rate was 0.72 with 106 records. Only one of the recessive TRD region (AR.18, Table 1; Reg.63, Table 4) showed a significant effect on heifer pregnancy, with a significant effect of − 0.115, reducing the pregnancy rate to 0.75, that is, 11% reduced the pregnancy rate. In addition, it is important to mention that those regions, found with a significant effect when comparing between AA × – and AB × AB matings, a reduced pregnancy rate was observed in the distribution of AA × AA and AB × – matings in some of these regions as well. In fact, their allelic TRD pattern anticipates that one single copy of the deleterious allele could generate a pregnancy loss and not only in the presence of the two copies (homozygous state). Finally, TRD regions that do not impact pregnancy rate are still important, as they potentially impact a different stage of the reproductive cycle, emphasizing the importance of investigating the consequences of all TRD regions.

**Table 4:** Effects of transmission ratio distortion (TRD) region on heifer pregnancy trait and distribution of pregnancy among at risk (both parents carrying the deleterious allele) and control matings.

Mating type	Effect on phenotype	P-value (FDR)	Sire × dam genotypes		Sire × maternal grandsire genotypes		Overall-TRD (Log_10_ (BF)	Additive-TRD (Log_10_ (BF)	dominance-TRD (Log_10_ (BF)
Region			AA × –(^c^)	AB × AB	AA × –	AB × AB			
Reg.50	− 0.166	0.00261 (0.104)	0.845 (68,255)	0.731 (26)	0.842 (75,358)	0.839 (137)	− 0.18 (534.69)	− 0.62 (164.73)	− 0.18 (75.93)
Reg.51 (AA.11)	− 0.093	0.02516 (0.541)	0.846 (69,091)	0.683 (41)	0.843 (75,965)	0.768 (69)	− 0.25 (1123.52)	− 0.98 (326.74)	− 0.26 (175.88)
Reg.52	− 0.267	0.00111 (0.053)	0.845 (70,273)	0.583 (12)	0.843 (76,533)	0.632 (19)	− 0.11 (64.05)	− 0.52 (52.47)	− 0.02 (− 1.16)
Reg.53	− 0.022	0.0096 (0.23)	0.847 (57,599)	0.828 (926)	0.846 (69,616)	0.841 (2629)	− 0.03 (70.85)	− 0.14 (77.79)	0 (− 2.43)
Reg.54	− 0.237	0.00086 (0.033)	0.845 (68,337)	0.444 (9)	0.843 (75,225)	0.717 (106)	− 0.11 (75)	− 0.52 (69.63)	− 0.01 (− 1.53)
Reg.55	− 0.108	0.022 (0.858)	0.847 (69,139)	0.75 (32)	0.843 (76,073)	0.783 (106)	− 0.08 (51.3)	− 0.41 (54.62)	0 (− 1.67)
Reg.56	− 0.07	0.00025 (0.009)	0.848 (66,135)	0.762 (172)	0.844 (74,058)	0.818 (379)	− 0.07 (95.03)	− 0.29 (72.75)	− 0.02 (− 0.56)
Reg.57	− 0.186	0.01644 (0.543)	0.846 (68,952)	0.583 (12)	0.843 (75,761)	0.714 (49)	− 0.43 (621.86)	− 0.88 (11.31)	− 0.79 (301.58)
Reg.58	− 0.073	0.00003 (0.001)	0.847 (65,165)	0.76 (283)	0.844 (73,738)	0.824 (688)	− 0.15 (968.32)	− 0.55 (428.56)	− 0.12 (90.57)
Reg.59	− 0.109	0.01698 (0.306)	0.846 (69,386)	0.743 (35)	0.843 (76,259)	0.796 (49)	− 0.01 (− 0.62)	− 0.39 (234.51)	0.18 (142.15)
Reg.60	− 0.028	0.00247 (0.081)	0.849 (59,018)	0.851 (858)	0.845 (69,406)	0.839 (2501)	− 0.08 (530.18)	− 0.28 (292.78)	− 0.06 (43.16)
Reg.61	− 0.109	0.03603 (0.829)	0.846 (68,912)	0.72 (25)	0.843 (75,893)	0.779 (86)	0.04 (19.03)	0.12 (15.76)	0.01 (− 1.74)
Reg.62	− 0.113	0.01334 (0.227)	0.846 (69,445)	0.771 (35)	0.843 (76,170)	0.804 (56)	− 0.16 (445.77)	− 0.72 (264.94)	− 0.08 (14.56)
Reg.63 (AR.18)	− 0.115	0.03141 (0.44)	0.847 (68,078)	0.75 (24)	0.844 (75,262)	0.791 (67)	− 0.01 (− 1.37)	− 0.5 (190.54)	0.24 (112.82)
Reg.64	− 0.178	0.00112 (0.025)	0.846 (68,977)	0.704 (27)	0.843 (76,029)	0.786 (56)	− 0.2 (338.44)	− 0.86 (158.42)	− 0.13 (19.24)

^a^Number of SNPs on the window; ^b^Parents’ genotypes; ^c^Number of matings, FDR: chromosome-wise false discovery rate.

## Conclusions

The analysis of a large genomic dataset allowed the characterization of TRD of the whole genome of Angus breed. Different parametrization uncovered 19 regions with recessive patterns (potential lethality for homozygote individuals) and 52 regions with allelic patterns. The allelic TRD discoveries exhibited complete or quasi-complete absence for homozygous individuals in addition to under-representation (potentially reduced viability) of carrier (heterozygous) offspring and also parent-specific TRD patterns. Using heifer pregnancy data, 64 and 20 regions showed significant effects at p-value < 0.05 and 0.01, reducing the progeny rate up to 15%. After chromosome-wise false discovery rate, the number of regions decreased to 12 and 4 at q-value < 0.05 and 0.01, respectively. The overlapping of TRD regions with recently published candidate lethal alleles in Angus supported the consistency of TRD findings. These novel findings in Angus present candidate genomic regions putatively carrying lethal and semi-lethal alleles providing opportunities to reduce the rates of embryonic losses or death of offspring as a way of improving fertility and fitness in beef cattle populations.

### Supplementary Information

Supplementary Information 1.

Supplementary Information 2.

## Data Availability

The datasets generated and/or analysed during the current study are not publicly available due to the agreements signed with the institutions but are available from the corresponding author on reasonable request.
